# A Double-Blind, Randomized Comparison Study between Zytux™ vs MabThera® in Treatment of CLL with FCR Regimen: Non-Inferiority Clinical Trial

**Published:** 2018-04-01

**Authors:** Gholamreza Toogeh, Mohammad Faranoush, Seyed Mohsen Razavi, Hassan Jalaeikhoo, Abolghasem Allahyari, Mohammad Reza Ravanbod, Fariba Zarrabi, Vahid Fallahazad, Ehsan Rezaei Darzi, Shadi Sadat Alizadeh Fard

**Affiliations:** 1 Thrombosis Hemostasis Research Center, Tehran University of Medical Sciences, Tehran, Iran; 2 Department of Internal Medicine, Hematology and Medical Oncology Ward, Imam Khomeini Hospital Complex, Tehran University of Medical Sciences, Tehran, Iran; 3 Pediatric Growth and Development Research Center, Institute of Endocrinology and Metabolism, Iran University of Medical Sciences, Tehran, Iran; 4 Clinic of Hematology and Oncology, Firoozgar Hospital, Iran University of Medical Sciences, Tehran, Iran; 5 AJA Cancer Research Center (ACRC), AJA University of Medical Sciences, Tehran, Iran; 6 Division of Hematology and Medical Oncology, Emam Reza Hospital, Mashhad University of Medical Sciences, Mashhad, Iran; 7 Department of Internal Medicine, Persian Gulf Tropical Medicine Research Center, Bushehr University of Medical Sciences, Bushehr, Iran; 8 Research Department, Mahak’s Pediatric Cancer Treatment and Research Center, Tehran, Iran; 9 Department of Epidemiology and Biostatistics, School of Public Health, Tehran University of Medical Sciences, Tehran, Iran; 10 School of Pharmacy, Shahid Beheshti University of Medical Sciences, Tehran, Iran

**Keywords:** Chronic lymphocytic leukemia, Rituximab, Zytux™, Mabthera^®^, Clinical trial

## Abstract

Background: Chronic lymphocytic leukemia (CLL) is characterized by accumulation of B cells in blood, lymphoid tissues and bone marrow. Addition of rituximab to CLL chemotherapy regimens has been associated with improved survival. The aim of this study was to establish efficacy and safety of Zytux™ in comparison to MabThera^®^ in treatment of CLL.

Materials and Methods: Seventy CLL patients who met the criteria for entering the study were randomized into two groups (35 patients in each group). Both groups received Fludarabine and Cyclophosphamide plus Rituximab as part of the FCR regimen. Group A was treated with Zytux™, and group B was treated with MabThera^®^. A non-inferiority margin of 20% for the primary outcome was defined to examine the similarity between Zytux™ and MabThera^®^.

Results: Baseline demographic characteristics showed no statistically signiﬁcant difference between the two groups.

The two treatment groups were comparable in terms of laboratory and clinical findings, cellular index changes and CD (5, 19, 20 and 23) counts during therapy cycles and at the end of the treatment period. Regarding safety results, Zytux™ demonstrated a similar profile of adverse reactions in comparison to MabThera^®^. Moreover, the overall response rate was 88% and 89% for Zytux™ and MabThera^®^, respectively (CI -0.17, 0.18).

Conclusion: Results showed non-inferiority of Zytux™ in terms of efficacy and adverse events as a biosimilar version of MabThera^®^.

## Introduction

 Chronic lymphocytic leukemia (CLL) is the most prevalent form of chronic leukemia ^1^^,^^2^. The course of the disease generally contains multiple relapses and remissions ^3^. Current therapies are more likely to lessen the symptoms, disease progression or the frequency of relapses rather than cure, and currently available chemotherapy regimens* need to be reinforced* with more efficacious novel therapeutic agents ^4^^-^^8^. 

Monoclonal antibodies including rituximab (that binds to CD20 antigen on B-cell surface) are among the most effective treatment options ^9^^,^^10^. In addition, a number of studies have revealed that the survival and cure rates were significantly higher in patients treated with Rituximab, Fludarabine and Cyclophosphamide (FCR) compared to Fludarabine and Cyclophosphamide (FC) alone and, among other various chemotherapy regimens, FCR is widely accepted as standard plan for treatment of CLL ^11^^-^^13^. Based on these findings, rituximab was approved for treatment of CLL at 500 mg/m^2^ in combination with FC regimen.

Monoclonal antibodies development requires multiple complex processes, and therefore these high-tech products are generally high-priced ^14^. In order to provide opportunities to improve healthcare access, expand outcomes and reduce costs, biosimilar agents (molecules similar in structure, function, and safety to the original biological drugs) are introduced ^15^.

Zytux™ (Rituximab, AryoGen Pharmed) is the intended biosimilar product of MabThera^®^ (Rituximab, Roche) which has exhibited acceptable compatibility profile during non-clinical phase. In order to show biosimilarity of Zytux™ to MabThera^®^, we have conducted a double-blind, randomized, non-inferiority clinical trial in CLL patients to compare the efficacy and safety of the two products. 

## MATERIALS AND METHODS

**Design**

This trial was designed with two parallel arms and random group assignment. The comparator medicine and the control were Zytux^™^ and MabThera®, respectively. Patients also received Fludarabine and Cyclophosphamide as part of the FCR regimen. Eligible patients were randomly assigned into two groups using a permuted-block randomization method at the end of the patients’ first visit, and the assessors were blinded to brand of rituximab administered (double- blind trial). A non-inferiority margin of 20% for primary outcome was selected by principal investigator (licensed oncologist) as an acceptable estimated difference between the comparator and the control. All procedures performed in studies involving human participants were in complete accordance with the 1964 Helsinki declaration and its later amendments and/or comparable ethical standards. The trial was approved by the ethics committee of Iranian Blood Transfusion Organization (IBTO) and was registered at Iranian Registry of Clinical Trials (IRCT201305296302N5).

**Patients**

The inclusion criteria were age of 18 years or more, diagnosis of untreated or recurrent B-CLL with indication for therapy (according to the National Cancer Institute Working Group diagnostic criteria), positive CD20 marker and Binet stage of B or C. Patients were excluded from study if they had one or more of following conditions: pregnancy or lactation, severe autoimmune hemolytic anemia, current active infections or underlying diseases such as Hepatitis B or C, HIV, severe cardiac or pulmonary disorders, recent myocardial infarction, uncontrolled hypertension and epilepsy, diabetes mellitus, elevated hepatic enzyme levels (more than 2 fold ULN), serum creatinine more than 2 mg/dl, known hypersensitivity with anaphylactic reaction to chimeric monoclonal antibodies or any of study drugs.

**Procedure**

Demographic information, medical history, physical examination, required lab tests (including CBC with differential, liver function tests, renal function tests, flow cytometric evaluation of CD counts and hemoglobin), especially disease staging were assessed during this visit. Subsequent visits were scheduled for chemotherapy administration; all efficacy and safety measures were then reassessed at each visit prior administration of chemotherapy regimen.

All patients received combination therapy of Fludarabine (25 mg/m^2^ IV infusion, on 2^nd^ to 4^th^ day of each cycle), Cyclophosphamide (250 mg/m^2^ IV infusion, on 2^nd^ to 4^th^ day of each cycles) and Rituximab (375 mg/m^2^ at first cycle and 500 mg/m^2^ at all subsequent cycles, IV infusion on day 1 of each cycle) as standard 28-day cycles. Group A was treated with Zytux™ and group B was administered MabThera^®^. It should be noted that due to took four courses of therapy during research protocol and two additional courses out of project.

**Outcomes**

The primary outcome was overall response rate (ORR) which is defined by the sum of complete response rate (CR) and partial response rate (PR). These were assessed following completion of scheduled chemo-immunotherapy cycles based on IWCLL (International Workshop on CLL) response criteria as shown in Table 1 ^16^. Secondary outcome measures were B-cell specific markers and safety profile (the comparison of complications and adverse reactions) which were evaluated during and upon completion of scheduled chemotherapy cycles.

**Table 1:** Definition of complete and partial response

**Parameter**	**Complete response ** **(all required)**	**Partial response**
Lymphocytes	≤4000/µl	≥50%↓^a^
Lymph Nodes	No palpable disease (LN<1.5cm)	≥50%↓ a
Splenomegaly	None	≥50%↓ a
Hepatomegaly	None	≥50%↓ a
Constitutional symptoms	None	Variable
Neutrophils	≥1,500/µl	≥1,500/µl or ≥50% improvement b
Platelets	>100,000/µl	>100,000/µl or ≥50% improvement b
Hemoglobin	>11g/dl (untransfused)	>11g/dl or ≥50% improvement b

a : must achieve at least two of parameters

b : must achieve at least one parameter

**Statistical analysis**

For Non-inferiority inference, normal approximation test was used to compare overall response as primary outcome between the two groups. Forest plot compared statistical confidence interval and clinical distance graphically. Next, generalized estimating equation models were applied to analyze antigenic outcomes according to time-to-event data. Frequency and type of adverse reactions were measured by descriptive tools. Laboratory parameters and baseline characteristics were analyzed by Mann-Whitney and t-test. Per-protocol-analysis was applied to analysis ideal patients. All statistical analyses were conducted using STATA 11. All *p*-values less than or equal to 0.05 were considered statistically significant.

## Results

**Patients**

**Figure 1:**
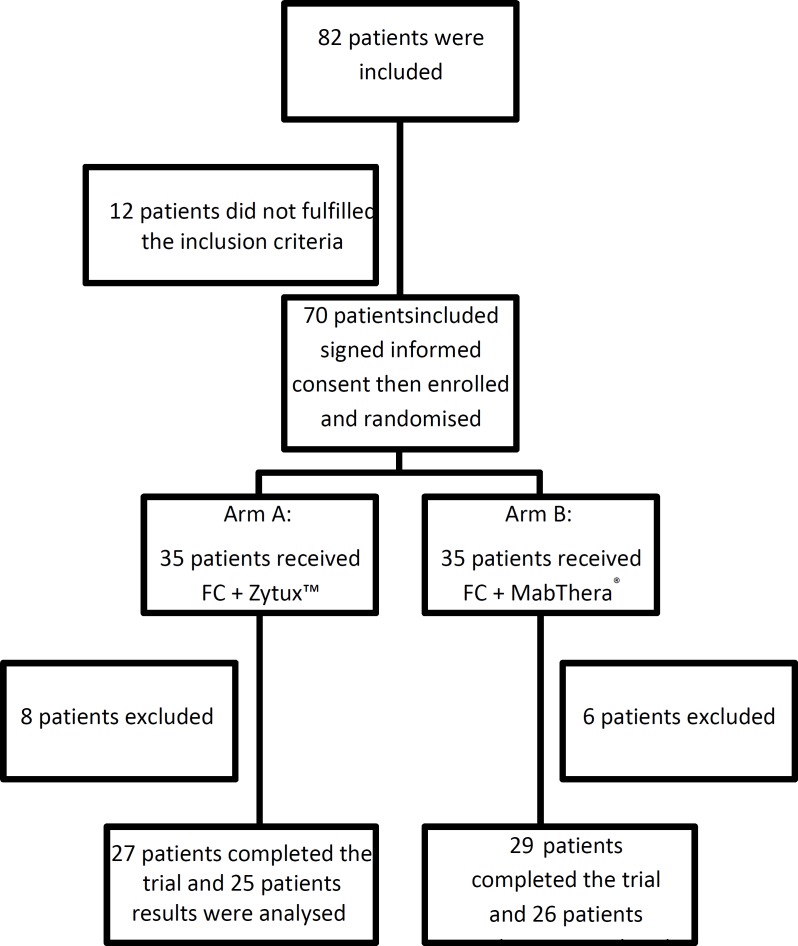
Trial Profile

A total of 82 patients were screened and 70 eligible patients were enrolled. As shown in Figure 1, 14 of the 70 patients randomized did not receive allocated intervention: 7 discontinued treatment (5 in Zytux™ arm and 2 in MabThera^®^ arm), 2 expired (both in MabThera^®^ arm) and 5 were lost to follow-up (3 in Zytux™ arm and 2 in MabThera^®^ arm). 

**Baseline information**

Baseline and demographic data analysis demonstrated adequate homogeneity between treatment arms. The mean±SD of age was 57.94±8.44 and 59.24±8.16 years for Zytux™ and MabThera^®^ groups, respectively. Majority of patients were males (28/7 male/female in Zytux™ group and 29/6 male/female for MabThera^®^ group). Baseline lab tests including WBCs, Plt, Hb, liver and renal function tests were comparable among treatment groups. Demographic information is summarized in Table 2. The difference between baseline information was considered insignificant. 

Moreover, disease staging assessment revealed that lymphadenopathy was present in 25 patients in Zytux™ arm and 26 patients in MabThera® arm (*p*-value=0.94), splenomegaly was present in 21 patients in Zytux™ arm and 25 patients in MabThera® arm (*p*-value=0.27) and hepatomegaly was present in 3 patients in Zytux™ arm and 5 patients in MabThera® arm (*p*-value=0.43).

**Table 2:** Baseline demographic data

**Variable**			**Zytux™ group (n=35)**	**MabThera® group (n=35)**
Baseline demographic data	Age (years)		57.94±8.44	59.24±8.16
	Sex (M/F)		28/7	29/6
	Weight (Kg)		71.06±11.40	69.45±11.47
	Body Mass Index (Kg/m^2^)		24.44±4.04	25.33±4.60
	Body Surface Area (m^2^)		1.83±0.16	1.78±0.16
Baseline laboratory data	WBC × 10^3^ (cells/mm^3^)		69.34±54.81	52.88±53.73
	Hb (g/dL)		12.05±2.18	12.20±2.45
	Plt × 10^3^ /microliter		128.63±66.55	139.40±52.54
	Lymphocyte %		84.07±14.56	77.67±13.27
	Reticulocyte × 10^6^ /microliter		0.89±0.39	0.82±0.46
	SGOT (units/Liter)		20.41±7.33	19.22±5.71
	SGPT (units/Liter)		16.48±6.92	15.64±4.76
	ALKP (units/Liter)		226.40±80.78	224.26±73.45
	Creatinine (mg/dL)		1.06±0.23	1.11±0.22
	Bilirubin (mg/dL)		0.77±0.36	0.68±0.35
Disease Staging	Binet stage	Stage B	22	24
		Stage C	12	10

**Primary outcomes**

The ORR (CR + PR) as the primary end point was comparable between the treatment groups (88% and 89% for Zytux™ and MabThera®, respectively)(Table 3).

**Table 3:** Clinical Response Rates

Response	Zytux™ group (n=25)	MabThera® group (n=26)	95% confidence interval	*p-value*
Overall	22 (88%)	23 (89%)	(-0.17,0.18)	0.96
Complete	15 (60%)	15 (58%)	(-0.29, 0.25)	0.87
Partial	7 (28%)	8 (31%)	(-0.22, 0.28)	0.83

**Secondary outcomes**

**Figure 2:**
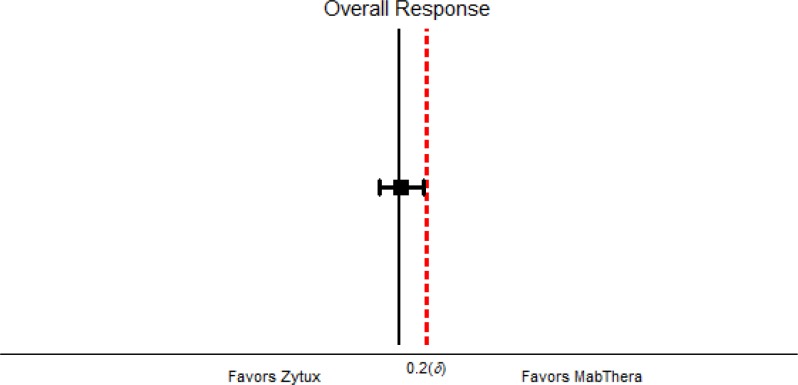
Non-inferiority Forest Plot

**Figure 3:**
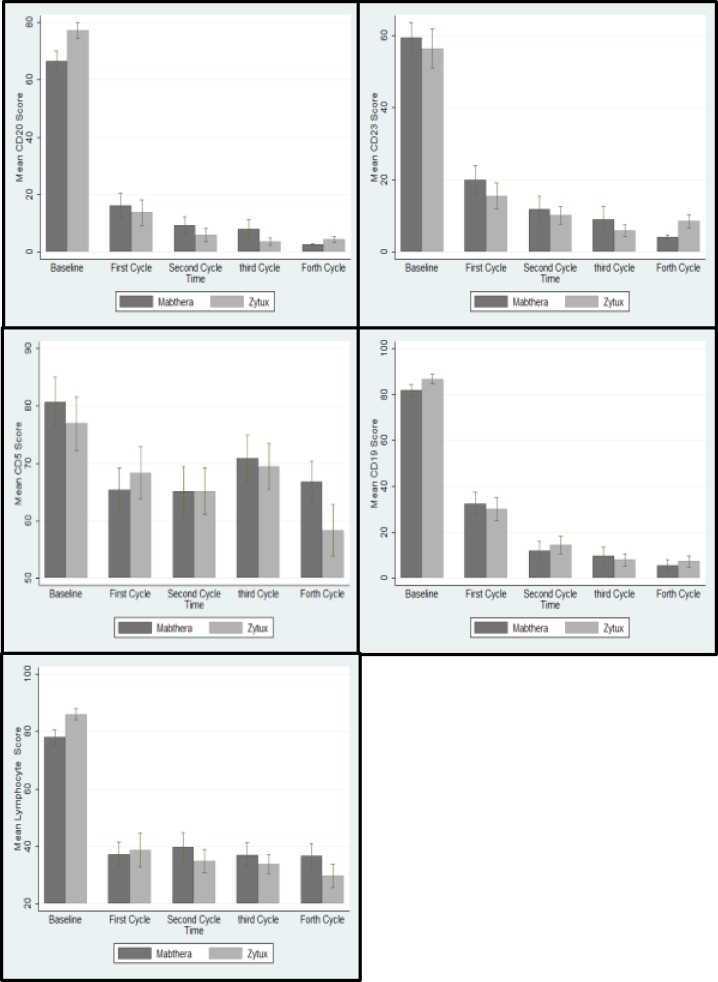
Lymphocyte count and CD markers after four cycles

B-cell specific CD markers of interest analysis showed no statistically significant difference between the treatment groups. Peripheral blood lymphocyte counts dropped off significantly from baseline through chemotherapy cycles and remained comparable between treatment groups at each cycle and upon treatment completion (Figure 2, Figure 3).

**Infusion reactions**

Incidence and type of infusion reactions occurred with Zytux™ were comparable to that of MabThera^®^ (26% vs 20% for Zytux™ and MabThera^®^, respectively). The incidence of infusion reactions at first cycle was slightly higher with Zytux™ compared with MabThera^®^ (26% vs. 14%). This trend did not achieve statistical significance and was not present at subsequent cycles (Table 4). The most frequent infusion reactions reported were shortness of breath (5 vs. 3), palpitation (3 vs. 3), rigors (3 vs. 3) and asthenia (3 vs.1) for Zytux™ and MabThera^®^, respectively.

**Table 4:** Incidence of infusion-related reactions

	**Infusion ** **Cycle**	**Zytux™ group ** **(n=35)**	**MabThera** ^®^ ** group ** **(n=35)**	**Total**
Incidence	First Cycle	9 (26%)	5 (14%)	14 (20%)
	Overall	9 (26%)	7 (20%)	16 (22.86%)

**Hematologic toxicities**

Summary of the hematologic toxicity analysis is presented in Table 5. As shown, incidence of grade I thrombocytopenia was slightly higher for MabThera^®^ group (non-significant); however, grade III and IV thrombocytopenia occurred more frequently in Zytux™ group. Seventy-nine events of any grade anemia were recorded, which were almost equally distributed in both groups. Thirty-three and 36 events of neutropenia were observed in Zytux™ and MabThera^®^ groups, respectively, although the rates of grade III/IV neutropenia were not significantly different between the two treatment groups.

**Table 5:** Hematologic adverse reactions

		**Zytux™ group** **(n=122)**	**MabThera** ^®^ ** group** **(n=115)**	**Total**	***p*** **-value**
Thrombocytopenia		No. (%)	No. (%)	No. (%)	
	Grade I (<LLN* to 75,000/mm^3^)	13 (11)	17 (15)	30 (13)	
	Grade II (50,000 to 75,000/mm^3^_)_	9 (7)	7 (6)	16 (7)	
	Grade III (25,000 to 50,000/mm^3^)	4 (3)	1 (1)	5 (2)	
	Grade IV (<25,000/mm^3^)	3 (2)	0 (0)	3 (1)	
	Total	29 (24)	25 (22)	54 (23)	0.18
Anemia (Hemoglobin level)					
	Grade I (<LLN* to 10 g/dL)	18 (15)	18 (16)	36 (15)	
	Grade II (8.0 to 10.0 g/dL)	18 (15)	16 (14)	34 (14)	
	Grade III (<8.0 g/dL)	5 (4)	4 (4)	9 (4)	
	Total	41 (34)	38 (33)	79 (33)	0.95
Neutropenia					
	Grade I (<LLN* to 1500/mm^3^)	11 (9)	13 (11)	24 (10)	
	Grade II (1000 to 1500/mm^3^)	17 (14)	16 (14)	33 (14)	
	Grade III (500 to 1000/mm^3^)	4 (3)	6 (5)	10 (4)	
	Grade IV (<500/mm^3^)	1 (1)	1 (1)	2 (1)	
	Total	33 (27)	36 (31)	69 (30)	0.92

* LLN: Lower Limit of Normal

**Non-hematologic toxicities**

Other adverse reactions observed in studied groups (other than infusion-related reactions) are presented in (Table 6). A total of 94 adverse reactions were observed during the scheduled chemotherapy cycles (47 in each group). The most prevalent adverse reactions were chills, nausea and hot flashes (observed 19, 13, and 8 times, respectively in total). There was one case of tumor lysis syndrome in MabThera^®^ group. Results of liver enzyme analysis showed an incremental trend following chemotherapy administration, which were slightly lower for Zytux™ treated group, but neither the differences nor the increment were statistically significant.

**Table 6:** Type of non-hematologic adverse reactions

Type	Zytux™ group (n=35)	MabThera^®^ group (n=35)	Total
Chills	7	12	19
Nausea	8	5	13
Hot flashes	4	4	8
Weight loss	4	1	5
Fever	2	2	4
Infection	3	1	4
Asthenia	2	1	3
Shortness of Breath	1	2	3
Pruritus	1	2	3
Other*	15	15	30

Other adverse reactions include reactions with occurrence of <5%.

## Discussion

 Based on efficacy and safety results of rituximab in different chemotherapy regimens, finding more available biosimilar medications could be helpful in reducing costs of therapy and enhancing more consequent treatments. New treatments for CLL are being developed, and targeted therapy is becoming more and more important. The role of CD20 inhibitors must not be overlooked as drugs such as ofatumumab and obinutuzumab are recently approved by the FDA ^17^^-^^22^. Data from recent studies demonstrated that FCR regimen may result in higher complete response rates and also more prolonged duration from treatment to relapse in CLL patients ^22^.

The primary outcome for which the non-inferiority margin was defined is ORR. The predefined margin for acceptable difference between treatment effects of Zytux™ and MabThera^®^ was 20%. As the results of this study confirmed the efficacy of Zytux™ in terms of ORR, it was shown to be non-inferior to MabThera^®^ by the defined margin (Figure 3). It is worth mentioning that the obtained results for ORR in our trial were in agreement with various studies in the literature in which the efficacy of rituximab was assessed along with FCR regimen and the response rate was cited 90-95% ^11^^, ^^22^^, ^^23^. 

As a secondary outcome, flow-cytometric assays on lymphocyte surface antigens of interest, including CD5, CD19, CD20 and CD22 demonstrated significant drop off in the percentage of cells expressing these antigens from baseline following administration of FCR regimen. The rates and magnitude of effects with Zytux™ resembled that of MabThera^®^, supporting the results obtained from the primary outcome. 

Infusion reactions are the most anticipated adverse reactions associated with rituximab administration, especially during the first cycle of treatment ^12^^, ^^20^^, ^^24^ with the most reported being flu-like symptoms, chills and fever. It has been reported that infusion reactions occur in more than 50% of patients at early stages of the first cycle of infusion, but decrease in subsequent cycles ^25^^-^^27^. The rates reported in this trial were remarkably lower than the literature, which could be due to exact implementation of infusion protocol and close monitoring applied in our setting. The most occurred reactions in the current study were chills, nausea and hot flashes.

Hematologic adverse reactions induced by chemotherapy regimens are of particular importance as they are directly associated with patient’s quality of life and treatment outcomes. Regarding these facts, the safety profile of biosimilar products concerning hematologic toxicities needs to be closely considered. The results of the current study demonstrated that there were no statistically or clinically meaningful diversity between Zytux™ and MabThera^®^ according to the hematologic toxicities. The hematologic events were in line with literature in terms of frequency and intensity ^1^^, ^^27^, and none of the events led to therapy discontinuation.

The non-hematologic adverse reactions seen in this study were generally mild, except for one case of tumor lysis syndrome which occurred in MabThera^®^ group, and also there was one case of hospitalization due to infection in Zytux™ group. In line with literature ^12^^, ^^20^^, ^^23^^, ^^24^^, ^^28^, the most common non-hematologic adverse reactions observed were chills, nausea, fatigue, pain and flu-like syndrome and, except for one case of tumor lysis syndrome, other events were mild to moderate, and no therapy interruptions were indicated. The profile of these reactions for Zytux™ were corresponding to that of MabThera^®^. 

The limitations of this study included small sample size and lack of survival information of patients because the complete follow-up was not performed. Furthermore, we only investigated the role of the biosimilar in CLL treatment. According to main guidelines, the efficacy or safety of biosimilar needs to be established in at least one of the indications to use it as a reference drug.

## CONCLUSION

 According to the results of the current study, Zytux™ (Rituximab, AryoGen Pharmed) administration has been associated with comparable outcomes in terms of efficacy and adverse events in comparison to MabThera^®^, and as a result the non-inferiority of Zytux™ to MabThera^®^ is established.
